# Identifying Modifiable Predictors of COVID-19 Vaccine Side Effects: A Machine Learning Approach

**DOI:** 10.3390/vaccines10101747

**Published:** 2022-10-19

**Authors:** Sara Abbaspour, Gregory K. Robbins, Kimberly G. Blumenthal, Dean Hashimoto, Karen Hopcia, Shibani S. Mukerji, Erica S. Shenoy, Wei Wang, Elizabeth B. Klerman

**Affiliations:** 1 Department of Neurology, Massachusetts General Hospital, Boston, MA 02114, USA; 2 Division of Sleep Medicine, Harvard Medical School, Boston, MA 02114, USA; 3 Department of Medicine, Division of Infectious Diseases, Massachusetts General Hospital, Boston, MA 02114, USA; 4 Harvard Medical School, Boston, MA 02114, USA; 5 The Mongan Institute, Massachusetts General Hospital, Boston, MA 02114, USA; 6 Division of Rheumatology, Allergy, and Immunology, Massachusetts General Hospital, Boston, MA 02114, USA; 7 Department of Medicine, Massachusetts General Hospital, Boston, MA 02114, USA; 8 Department of Medicine, Brigham and Women’s Hospital, Boston, MA 02114, USA; 9 Occupational Health Services, MassGeneralBrigham, Boston, MA 02114, USA; 10 Department of Neurology, Division of Neuroimmunology and Neuro-Infectious Diseases, Massachusetts General Hospital, Boston, MA 02114, USA; 11 Infection Control Unit, Massachusetts General Hospital, Boston, MA 02114, USA; 12 Departments of Medicine and Neurology, Brigham and Women’s Hospital, Boston, MA 02114, USA

**Keywords:** vaccination, COVID-19, side effects, allergy, time-of-day-effects, machine learning, model explanation

## Abstract

Side effects of COVID-19 or other vaccinations may affect an individual’s safety, ability to work or care for self or others, and/or willingness to be vaccinated. Identifying modifiable factors that influence these side effects may increase the number of people vaccinated. In this observational study, data were from individuals who received an mRNA COVID-19 vaccine between December 2020 and April 2021 and responded to at least one post-vaccination symptoms survey that was sent daily for three days after each vaccination. We excluded those with a COVID-19 diagnosis or positive SARS-CoV2 test within one week after their vaccination because of the overlap of symptoms. We used machine learning techniques to analyze the data after the first vaccination. Data from 50,484 individuals (73% female, 18 to 95 years old) were included in the primary analysis. Demographics, history of an epinephrine autoinjector prescription, allergy history category (e.g., food, vaccine, medication, insect sting, seasonal), prior COVID-19 diagnosis or positive test, and vaccine manufacturer were identified as factors associated with allergic and non-allergic side effects; vaccination time 6:00–10:59 was associated with more non-allergic side effects. Randomized controlled trials should be conducted to quantify the relative effect of modifiable factors, such as time of vaccination.

## 1. Introduction

COVID-19 vaccines have been distributed to billions of individuals worldwide and have reduced serious illness, hospitalizations, and death [1]. As of July 2022, only 61% of the world’s population has been fully vaccinated against COVID-19 [2]. An important factor for vaccine hesitancy is concern about vaccine safety, efficacy, and side effects [3,4]. Understanding risk factors for vaccine-related side effects—especially ones that may be modifiable—is important for clinicians, for patient safety, for patient expectations and planning, and possibly for reducing hesitancy to be vaccinated.

At MassGeneralBrigham (MGB), a large integrated healthcare system, surveys were used to obtain information about post COVID-19 vaccination symptoms through email, text message, phone, and smartphone application links as part of employee health monitoring. Several publications using epidemiological analytic techniques have reported important clinical information using this dataset, including the incidence of allergic and non-allergic side effects after mRNA COVID-19 vaccination and their association with variables such as demographic characteristics, allergy history, and prior known infection with SARS-CoV-2 [5,6,7,8,9,10,11,12]. This report explores new potential covariates, including time-of-day for COVID-19 vaccinations (which is an easily modified factor), and utilized a different analysis approach—Machine Learning (ML).

Age, sex, race, hormones, and body mass index (BMI) have previously been associated with vaccination-related adverse effects for different vaccines [13,14,15]. We studied time-of-day of vaccination as a potential predictor because of the well-known impact of circadian rhythms on physiology [16] including immune responses [16,17] and because it is relatively easily modified. Effects of time-of-day of COVID-19 vaccination on anti-Spike antibody responses have been documented: late afternoon vaccination, being female and being younger were associated with higher response [18]. Different side effects related to time-of-day of other vaccines have also been reported [19,20].

ML is a powerful technique for diagnosis, detection, prediction, and prognosis in medicine. Studies have used ML-based approaches to explain the contribution of different variables (e.g., age, tumor size, and number of removed lymph nodes) in prediction of 10-year overall survival of breast cancer [21], identify the most critical factors in predicting the prevalence of stroke [22], and predict the risk of hypoxemia during general anesthesia and provide explanations of the risk factors (e.g., age, sex, BMI, blood pressure, temperature, and medication) [23].

In this study, our aim was to identify predictors of post COVID-19 vaccine-related side effects using ML methods on self-reported side effects for 3 days after the first vaccination of an mRNA-based vaccine.

## 2. Materials and Methods

### 2.1. Data

The dataset was created from MGB electronic health record’s COVID-19 Datamart and a REDCap (Research Electronic Data Capture [24]) survey that collected self-reported symptoms for 1–3 days after each vaccination. Detailed information about the REDCap side effect survey have been previously reported [5]. For this report, we only used data from the first vaccination.

Data from individuals were included if they (i) received an mRNA COVID-19 vaccine at a MGB site between December 2020 and April 2021, (ii) responded to at least one day of the REDCap survey, (iii) had documented time-of-day of administration of COVID-19 vaccine and (iv) did not have a COVID-19 diagnosis or positive polymerase chain reaction test within one week after the vaccination (because of overlap of disease and side-effect symptoms [5]). Individuals who received the Janssen adenovirus-based vaccine were excluded from this analysis for two reasons: only 1486 (<3%) individuals who met our inclusion criteria received a Janssen vaccine, and people with high risk of allergic reaction were recommended for the Janssen vaccine and this could introduce a bias in our dataset [25].

Variables considered as potentially associated factors for side effects were: demographics (age, sex (Female or Male), race (Asian, White, Black, or Other Race (i.e., Unknown/Missing, Other, Two or More, Declined, American Indian or Alaska Native, Native Hawaiian or Other Pacific Islander)), and ethnicity (Hispanic, Non-Hispanic, or Other Ethnicity (i.e., Unknown/Missing, or Declined))); any note of Epinephrine Autoinjector Prescription (e.g., EpiPen); any history of allergy (divided into Food, Vaccine, Medication, Insect sting, Seasonal, Latex and Other categories using coded and free-text data within the electronic health record allergy list); any COVID-19 diagnosis/positive test prior to vaccination; vaccine manufacturer (Pfizer: New York, NY, USA or Moderna: Cambridge, MA, USA); and clock time of vaccine administration and/or appointment. The number of different allergy history categories for an individual was summed. The variables considered for analysis were selected based on expert knowledge, feature selection approaches, and their availability in the database.

Outcomes of interest were side effects queried at days 1, 2 and 3 post-vaccination by a REDCap survey. The questions and response options were:

- Allergic symptoms: (Yes/No) (i) Rash or itching; (ii) Hives; (iii) Swollen lips, tongue, eyes, or face; (iv) Respiratory symptoms (wheezing, chest tightness, or shortness of breath).
- Non-allergic symptoms: (None/lower severity/higher severity) (i) New headache; (ii) New fatigue; (iii) Joint pain; (iv) Muscle pain; (v) Fever.

This study was approved by the MGB human research committee and MGB Occupational Health Services.

### 2.2. Pre-Processing

Race and ethnicity were grouped into 4 categories to reduce the number of categories tested: White/Non-Hispanic, Non-White/Non-Hispanic, Any-Race/Hispanic, and Any-Race/Other-Ethnicity. For time-of-day of vaccination, there were two data entries available: immunization time and appointment time. Immunization time was used preferentially; appointment time was used only if immunization time was not available. Time-of-day groupings for these analyses were 6:00–10:59, 11:00–15:59, and 16:00–21:59. Individuals receiving vaccines before 6:00 and after 22:00 were excluded because of low numbers (10 participants total).

To prepare the input features for analysis, categorical variables were converted into dummy variables using OneHotEncoder, a scikit-learn (version 1.0.1) preprocessing package in Python. To avoid collinearity effect between the input variables, a Variance Inflation Factor analysis was conducted (threshold = 5) [26].

To increase the classification performance, dimensionality reduction in the feature set is often necessary. In this study, two feature selection methods were applied: Shapley Additive exPlanations (SHAP) feature importance values (details below), and forward feature selection and backward feature elimination. Forward feature selection and backward feature elimination consist of adding features one by one to the feature set. If an added feature produced higher accuracy rate, it would stay in the feature set; otherwise, it would be removed. Once all features were evaluated, features in the obtained feature set were removed in inverted order if their subtraction did not negatively affect accuracy. After obtaining SHAP feature importance values from the ML Extreme Gradient Boosting (XGB) model, we chose the 8 best ranked features, since using more features did not improve our model’s performance (specifics below).

For allergic symptoms, if the response to an allergic symptom for any of the 3 days was “yes”, it was grouped as class 1 (=yes). If the response to an allergic symptom for all of the 3 days was “no”, then it was grouped as class 0 (=no). If a participant completed the survey for only one or two days and the responses on those days were both “no”, that entry was removed, because we do not know if the response on the missing day would have been “yes”. For non-allergic symptoms, similar logic was used to group as class 1 (=higher severity) or class 0 (=none/lower severity) or removal of entry.

### 2.3. Machine Learning Model

XGB, a tree-based ML model, was selected because of its execution speed and performance [21], high interpretability, and the possibility of identifying the strongest predictors by applying a model explanation such as TreeExplainer [26]. XGB (with max_depth = 3, number of estimators = 50, and learning rate = 0.1) was applied to predict any allergic (yes vs. no) and any non-allergic (higher severity vs. none and lower severity) side effects reported for 3 days after the vaccination. The model was parametrized using a randomized search of different parameter settings with a 5-fold cross validation. Since the dataset is not balanced in respect to the dependent variables, up sampling (RandomOverSampler an imbalanced-learn (version 0.7.0) over_sampling package in Python) was used to increase the number of samples for the minority classes (i.e., the yes responses to allergic symptoms and the higher severity responses to non-allergic symptoms) in the training sets.

### 2.4. Evaluation

A stratified k-fold (k = 5) cross-validation was used to validate the performance of the ML model. This method uses a large part of the data (80% of the data) to train the model, and a small part of the data (20% of the data) to test the model. The stratified cross-validation was repeated 10 times and the average and the standard deviation (SD) of F-score (Equation (1)) was calculated [27]. This evaluation metrics has a range of 0 to 1, a higher value shows a better performance.

(1) $$F-score=2\times \frac{precision\times recall}{precision+recall}$$

### 2.5. Explainability

One concern about ML is that the results are “black box” and not interpretable. To address this, we chose TreeExplainer that uses SHAP values, a game theory method for assigning an importance value to variables based on their contribution to the model [26], and to explain the magnitude and direction of the contribution of each of the variables to the model prediction [21]. This property therefore allows providing both new insights into the model’s variables and the relations between them. SHAP values were generated using the SHAP package (version 0.39.0 in Python). These values were used to obtain a visualization of the overall feature importance for the model. Then, to show how each variable contributed to the model’s output, we generated SHAP boxplots (by applying a seaborn (version 0.11.2) boxplot package in Python) for categorical variables (e.g., sex, race/ethnicity) and SHAP scatter plots for continuous variables (e.g., age). We also used SHAP’s local explainability feature (SHAP Waterfall plot) to display the effect of each of the variables for individual predictions.

**Figure 1:**
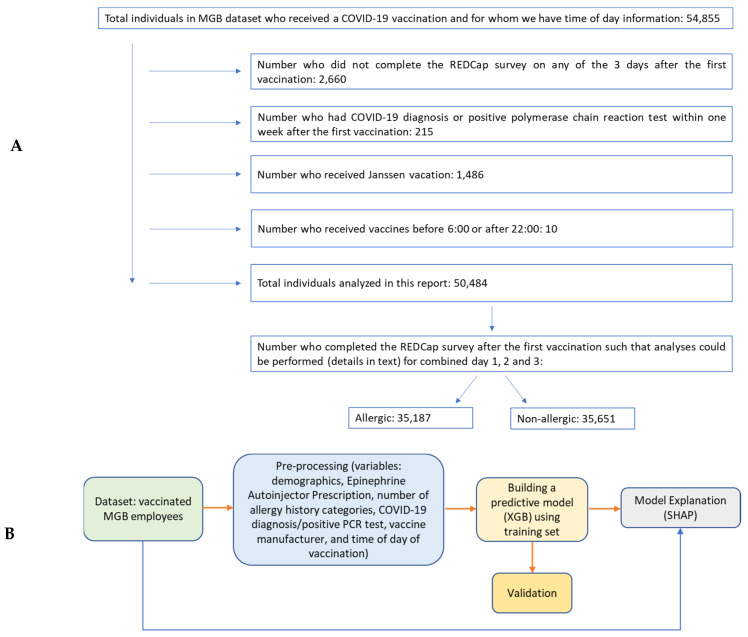
(**A**): Creation of dataset; (**B**): Block diagram illustrating the processing stages used in this study to identify predictors of post COVID-19 vaccine-related side effects.

The processing stages designed for this study are illustrated in Figure 1A,B. All analyses were performed using open-source libraries in Python 3.7.

## 3. Results

Data analyzed were from 50,484 individuals (Table 1, Figure 1A). Of these individuals, 60% received the Moderna vaccine; 73% were female; ages ranged from 18 to 95 years old; 8% had prior COVID-19 diagnosis or positive test. A total of 2% had Epinephrine Autoinjector Prescription; 28% had any history of allergy documented; and 34% received their vaccine from 6:00 to 10:59, 44% from 11:00 to 15:59 and 22% from 16:00 to 21:59. When both immunization and appointment time were available, the number of times for whom there was difference in immunization vs. appointment time group (i.e., 6:00–10:59, 11:00–15:59, 16:00–21:59) was 2.6% (156 of 6011 appointments).

Medical conditions including any history of thrombosis, myocardial infarction, or stroke were variables that did not show any impact on the model’s accuracy, and therefore, were not included in the final feature set. Eight variables/features (age, sex, race/ethnicity, Epinephrine Autoinjector Prescription, number of allergy history categories, any prior COVID-19 diagnosis or positive test, vaccine manufacturer, and time-of-day of vaccination) were used to build two predictive ML models; one for predicting any allergic symptoms (yes vs. no) and one for predicting any non-allergic symptoms (higher severity vs. none/lower severity) reported for 3 days after vaccination. The models showed predictive F-score values of 84% (SD = ±0.01) for allergic symptoms and 81% (SD = ±0.01) for non-allergic symptoms.

**Figure 2:**
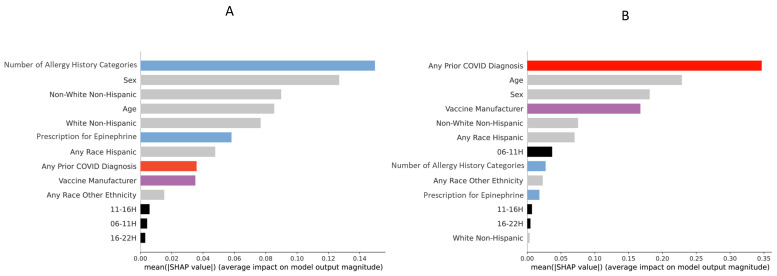
Feature importance plot using the mean absolute SHAP values for (**A**): allergic side effects and (**B**): non-allergic side effects. Colors: grey—demographics, blue—allergy history category/prescription for Epinephrine, red—any prior COVID diagnosis or positive test, purple—vaccine manufacturer, black—time-of-day of vaccination.

A SHAP feature importance plot was created using mean absolute SHAP values of the ML model for predicting allergic symptoms (Figure 2A): this plot orders the input variables (top to bottom along the *y*-axis) according to their importance to the ML model. The most important predictors to predict any allergic symptoms were, in descending order: number of allergy history categories, sex, race/ethnicity, age, Epinephrine Autoinjector Prescription, any prior COVID-19 diagnosis or positive test, vaccine manufacturer, and time-of-day of vaccination (Figure 2A).

**Figure 3:**
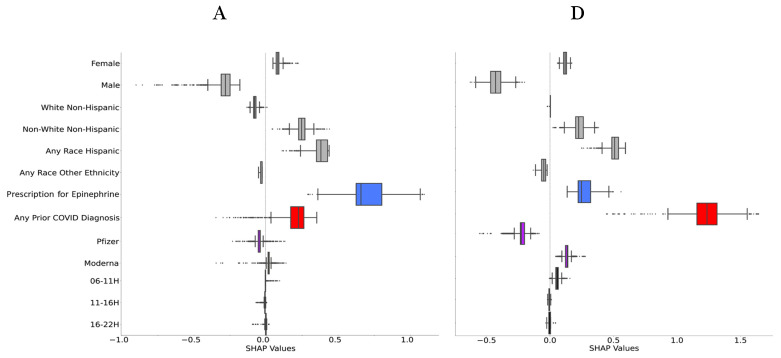

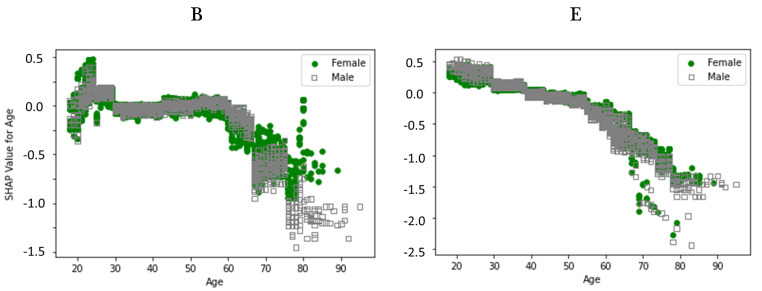

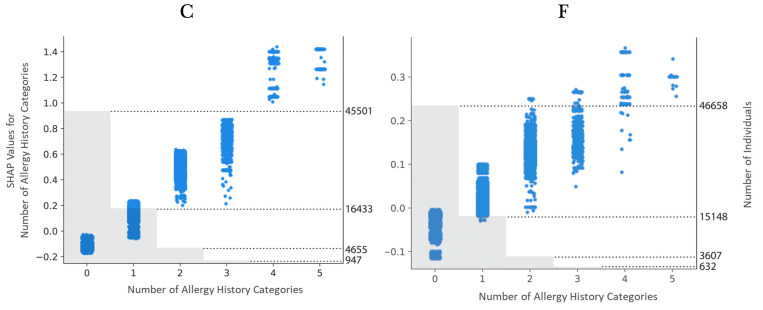
(**A**): SHAP value boxplot that shows the direction of impact of each variable on model’s output for allergic side effects. Positive SHAP values are indicative of having side effects, while negative SHAP values are indicative of not having side effects. Box limits indicate 25th and 75th percentile, vertical line within the box indicates 50th percentile, and other vertical lines indicate 5th and 95th percentiles; (**B**): SHAP feature independent plot for age vs. sex showing the impact of age on model output and (**C**): SHAP value scatter plot for number of allergy history categories showing the impact of this variable on model output. (**D**–**F**): as in (**A**–**C**) except for non-allergic side effects. Colors in (**A**,**D**): grey—demographics, blue—prescription for epinephrine, red—any prior COVID diagnosis or positive test, purple—vaccine manufacturer, black—time-of-day of vaccination.

The SHAP boxplot (Figure 3A) shows the direction of impact of the categorical variables on the model output in predicting report of allergic symptoms for 3 days after vaccination. Positive SHAP values are associated with a higher likelihood of reporting symptoms and negative SHAP values are associated with a lower likelihood of reporting symptoms. Females, Non-White/Non-Hispanic, Any-Race/Hispanic, Epinephrine Autoinjector Prescription, those who had any prior COVID-19 diagnosis or positive test, and people who received the Moderna vaccine were more likely to report allergic symptoms. No significant (i.e., the SHAP value of 0 was within 5–95% distribution of values) effect of time-of-day was found for allergic symptoms. For the continuous variable of age, younger adults of both sexes were more likely to report allergic side effects (Figure 3B), with magnitude approximately constant for ages 30–60 and then a decline in likelihood starting at ~60 years of age. There was a monotonically increasing effect of number of allergy history categories on the likelihood to report allergic side effects (Figure 3C).

For any non-allergic symptoms, the most important predictors in descending order of magnitude were any prior COVID-19 diagnosis or positive test, age, sex, vaccine manufacturer, race/ethnicity, time-of-day of vaccination, number of allergy history categories, and Epinephrine Autoinjector Prescription (Figure 2B). For non-allergic symptoms, in addition to the factors significant for allergic symptoms, significant effects of time-of-day (morning vaccinations (6:00–10:59) were also associated with more non-allergic side effects (Figure 3D); there was also a more linear effect of age on likelihood of non-allergic symptoms (Figure 3E). A lower magnitude of the monotonically increasing effect of the number of allergy history categories was also seen (Figure 3F).

**Figure 4:**
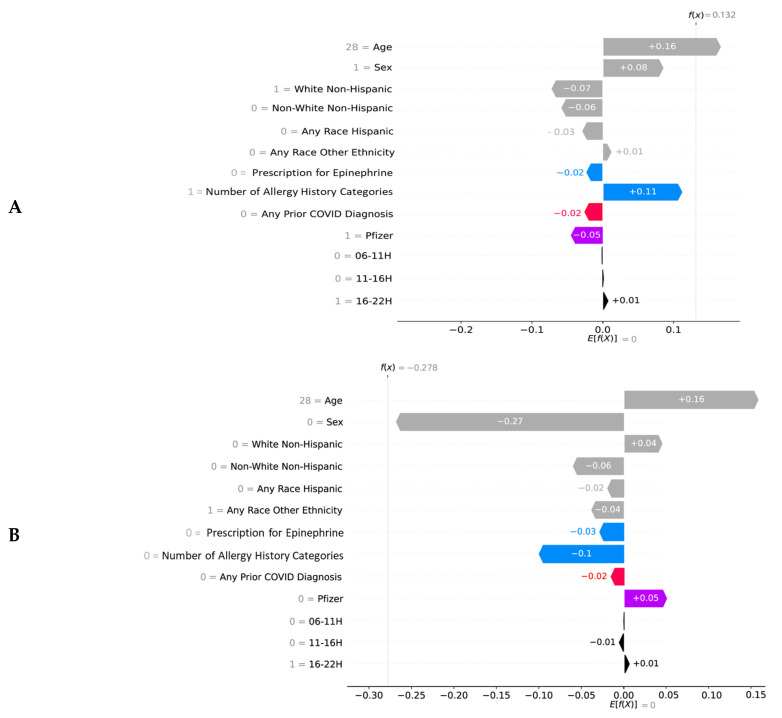

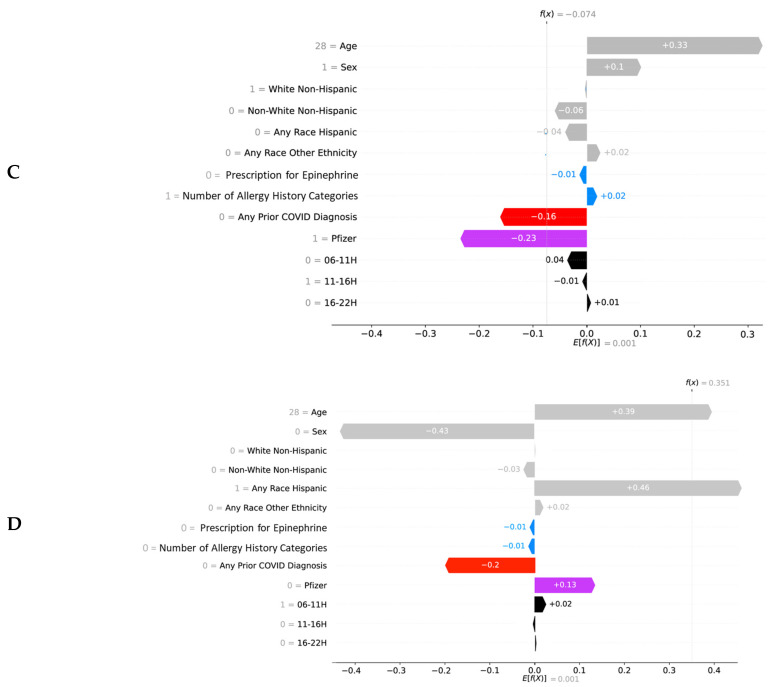
SHAP Waterfall plots exampling local/individual predictions for 4 individuals showing the contribution of each variable to the prediction. The gray text in front of each variable name is the value of the particular variable. The baseline value (E[f(X)]) is displayed below the *x*-axis, indicating the expected value of the model. The model output for each individual (f(x)) is shown on top of each panel; it is the sum of SHAP values calculated for all variables. Positive SHAP values push the model to predict having side effects, while negative SHAP values push the model to predict no side effects. Allergic side effects: (**A**): a 28 year old white Non-Hispanic male who received Moderna between 16 and 21:59. (**B**): a 28 year old male with any race Other Ethnicity who received Moderna between 16 and 21:59; Non-allergic side effects: (**C**): a 28 year old white Non-Hispanic female who received Pfizer between 11 and 15:59, and (**D**): a 28 year old male with any race Hispanic who received Moderna between 06 and 10:59. Absolute SHAP values < 0.01 were not presented on the figures. Colors: grey—demographics, blue—allergy history category/prescription for epinephrine, red—any prior COVID diagnosis or positive test, purple—vaccine manufacturer, black—time-of-day of vaccination.

SHAP values can also be used to create a “local” explanation for every observation/individual in the dataset (in addition to the global effects detailed above). To illustrate a local explanation for specific individual predictions, Waterfall plots were used: Figure 4 presents four examples. Each row in SHAP Waterfall plot shows the positive or negative contribution (*x*-axis) of each input variable (*y*-axis) to the overall likelihood of having allergic (Figure 4A,B) or non-allergic side effects (Figure 4C,D). For example: (i) characteristics such as being young (Age = 28), female, Hispanic, with a history of allergy, receiving Moderna and being vaccinated 6:00 to 10:59 and 16:00 to 21:59 (06-11H = 1, 16-22H = 1) increase the chance of having side effects after vaccination (ii) characteristics such as being male, White Non-Hispanic, any race other ethnicity, no history of Autoinjector Epinephrine Prescription, no history of allergy, no prior COVID diagnosis or positive test, receiving Pfizer, and being vaccinated between 11 to 15:59 (11-16H = 1) decrease the chance of having side effects after vaccination.

## 4. Discussion

We used an explainable ML method to identify predictors of post-COVID-19 vaccine side effects in a large dataset. Our results are consistent with several recent publications [5,6,8,9] that documented both non-allergic and allergic type side effects after COVID-19 vaccination and identified the effect of different factors influencing the severity of reported side effects after COVID-19 vaccinations. These findings include: (i) association of reported non-allergic symptoms after vaccination with demographic characteristics and prior COVID-19 diagnosis or positive test [5,8]. We also documented that female, younger individuals, Non-White race, Hispanic ethnicity, and those with prior COVID-19 infection were more likely to report non-allergic side effects after vaccination. (ii) association of reported allergic reactions to mRNA COVID-19 vaccines with a history of allergic reaction [6] (iii) the Moderna vaccine is associated with more allergic reactions than the Pfizer vaccine.

We also identified previously unreported factors affecting side effects reported after the first dose of an mRNA COVID-19 vaccine: number of allergy history categories, a history of Autoinjector Epinephrine Prescription and the modifiable factor, time-of-day of vaccination. Circadian rhythms are physical, mental, and behavioral changes that display a period of approximately 24 h [28]. These rhythms influence almost all areas of physiology, including the sleep-wake cycle, body temperature, blood pressure, and heart rate [16]. Chronomedicine aims to incorporate knowledge of biological rhythms to increase treatment effectiveness, including reduction in side effects. Timing the administration of a drug to coincide with peak levels of its physiologic target has shown clinical benefits in hypertension, hypercholesterolemia, cancer, and other areas [29]. Currently, health care professionals rarely consider time-of-day in their diagnosis and treatment administration [30] and often vaccination times are chosen by convenience. The information about time-of-day may be used to better define relevant physiology (i.e., the multiple components of a response to vaccination, some of which may differ by time-of-day) and improve clinical care. For example, altering the time of COVID-19 vaccination to lower unwanted side effects would be a relatively low-cost and scalable change in practice.

Electronic health data provide the opportunity to improve healthcare. Handling these large and complex datasets requires special computational techniques that can deal with these datasets. ML techniques have broad applications in healthcare and are helpful in identifying patterns in large datasets [31]. Developments in the area of ML and model explanation, and strong methods to compute and visualize the magnitude and direction of impact of input variables on model’s outputs, can help translate knowledge from science to practice [26,32]. Given our multidimensional datasets, the application of ML can be useful since its strength includes dealing with many input variables.

Limitations of this work are that the data are from an observational study. Randomized clinical trials should be performed to further test our hypotheses of time-of-day effects. Collecting time-of-day of vaccination data and data about sleep obtained before or after the vaccination [33,34,35,36] should be included in future studies. Future work should also (i) explore side effects occurring during the 3 days after the second dose using these techniques: (ii) the impact of night shift work before and/or after COVID-19 vaccination on self-reported side effects, and (iii) target underlying physiological reasons.

## 5. Conclusions

In this study, we used XGB, a ML model to predict the occurrence of self-reported COVID-19 vaccination side effects using a range of variables (e.g., demographics, history of allergy, vaccine manufacturer and time-of-day of vaccination). We then used a model explanation technique (SHAP) to identify the important predictors of COVID-19 vaccine-related side effects and explain the effect of the input variables on model’s output. Our results demonstrate that demographics, any history of allergy, any prior COVID diagnosis or positive test, vaccine manufacturer, and time-of-day-of-vaccination (6:00–10:59 associated with significantly more non-allergic side effects) effects on side effects reported for three days after the first dose of a COVID-19 vaccination. This information can be used to understand the risk factors of adverse events and for planning of possible time-out of work for healthcare workers and patients (e.g., reduce risk for needing to miss work after vaccination).

## Figures and Tables

**Table 1:** N: number of individuals and (%) percent of total participants.

Variables	N	(%)
Age in Years		
Age Group 1 (18–40)	25,213	50
Age Group 2 (41–60)	18,529	37
Age Group 3 (61–95)	6742	13
Total	50,484	100
Sex		
Female	36,801	73
Male	13,683	27
Total	50,484	100
Race/Ethnicity		
White/Non-Hispanic	28,408	56
Non-White/Non-Hispanic	8066	16
Any Race/Hispanic	2662	5
Any Race/Other Ethnicity	11,348	23
Total	50,484	100
Prescription History		
Epinephrine Autoinjector Prescription	1246	2
Allergy History		
Any History of Allergy	14,197	28
COVID-19 Diagnosis/Positive PCR Test		
Any Before Vaccination 1	3797	8
Vaccine Manufacturer		
Pfizer	20,324	40
Moderna	30,160	60
Total	50,484	100
Clock Time of Vaccine Administration/Appointment		
Time 1 (6:00–10:59)	17,254	34
Time 2 (11:00–15:59)	22,367	44
Time 3 (16:00–21:59)	10,863	22
Total	50,484	100

## Data Availability

The data are not available to external parties.
